# 

**Published:** 1921-05-28

**Authors:** 


May 28, 1921] [Price Twopence

### The Hospital

The Workers' Newspaper of Administrative Medicine and Institutional Life, Administration, National Insurance and Health.

CONTENTS

**Industrially Employed Mothers** ... ... ... 141
**The Hospital Pageant** ... ... ... ... ... 141
**The Hospital Pageant Meeting** ... ... ... 142
General Agreement.
**Notes of the Week** ... ... ... ... ... ... 143
The Prince at Bristol—A Long Run of Legacies—Death of Mr. Walter Alvey—Crowded Universities—The General Medical Council—Egg Poisoning—Thomas Guy—Dangerous Drugs—The Control of Fevers—The Value of Minor Allies—The Eastbourne Hospital Inquiry—A Criticism of Co-operative Buying—Institutional Poaching.
**A Note on Overclothing** ... ... ... ... ... 145
Customs, Fashions and Colds.
**The Profession and the Ministry** ... ... ... 146
**X-ray and Radium Protection Committee** ... 146
**Extemporised Sanitation** ... ... ... ... 147
The Best Methods for Newly Claimed Areas.
**Annual Meetings of Hospitals** ... ... ... 148

CONTENTS

**The Public Well-Being** ... ... ... ... ... 149
The Typhus Situation—"Saving" Half-a-Million—Lectures for Mothers—Happy Old Age.
**The Institutional Library** ... ... ... ... 151
Standard Nomenclature of Diseases and Pathological Conditions, Injuries, and Poisonings, for the United States—Essentials of Medicine: a Text-book of Medicine—The Medical Register, 1921. The Dentists' Register, 1921—Clinical Disorders of the Heart Beat.
**From the Patient's Point of View** ... ... ... 152
**The Matrons' and Sisters' Department** ... ... 153
The Year's Work of the College of Nursing.
**Round the Hospitals** ... ... ... ... ... 153
**The Royal British Nurses' Association Club** ... 155
**Central Midwives Board for Scotland** ... ... 156
**Conferences at the Nursing and Midwifery Exhibition** ... ... ... ... ... 157
**Matrons' and Sisters' Appointments** ... ... 158

